# The Croonian Lectures, Delivered at the Royal College of Physicians, in 1833, on Cholera

**Published:** 1834-04-01

**Authors:** 


*The Croonian Lectures, delivered at the Royal College of Physicians, in* 1833, *on Cholera.* By GEORGE LEITH ROUPELL, M.D. —London, 1833. 8vo. pp. 91.

WHEN we open a book on cholera, it is almost with despair of being much the wiser for its contents: the obscurity, however, which pervades this subject, only augments the merit of every well-directed attempt to dispel it, and all contributions from those whose experience and judgment entitle them to be heard have a right to the careful attention of the profession. Dr. Roupell has enjoyed considerable opportunities of witnessing the disease of which he writes, and has stated the results of his experience in a judicious, practical, and unostentatious manner.

We quite agree with him in considering the malignant cholera as a disease of modern origin; he correctly states, however, that it was certainly known as early as the year 1782. Mr. Curtis's description, quoted from the Madras Reports, puts it beyond a doubt that the disease prevalent in Sir Edward Hughes's squadron, and believed to have arisen from communication with an infected port in Ceylon, was in all respects identical with that which, in its epidemic form, has since devastated the East, and spread over the greater portion of the globe. Our author adds,

"Mr. Barnes, who was the medical superintendent at Jessore from the year 1810, mentions, in a letter addressed to me as one of the medical officers of the City board of health, that he had become acquainted with the disease since he had joined the station; that it was to him entirely new, and one which had superseded the periodical remittent fever, formerly endemic. He states that, twice previously to 1817, this disease prevailed to such an extent in Jessore, and its immediate neighbourhood, as to render it necessary to shut the courts of justice, and suspend all business, for a considerable length of time. The origin of this monstrous birth may then be dated earlier than the period usually assigned to it. Its origin, too, as an epidemic may be ascribed to other places than Jessore, as Dr. Christie relates that it existed in the various parts of the southern districts of Bengal, in the months of May and June, 1817; that it did not create alarm in Jessore till August; that several cases took place in Calcutta on the 5th of September, and from that day forward the disease became daily more frequent." (P. 16.)

After a judicious review of some of the principal circumstances which have been supposed to constitute the remote causes of this singular pestilence, Dr. Roupell justly concludes that we are still entirely in the dark upon the subject, nor less so on the causes which favour or impede its epidemic extension.

In intimating his own conviction of the *contagious* nature of the disease, he expresses himself with becoming candour. It appears to us that this question must for the present remain open. The evidence hitherto brought on either side is altogether inconclusive: the apparent importation of the disease from places where it is prevalent, its appearance on board vessels shortly after their leaving port, and other circumstances on which the doctrine of contagion has been made to rest, admitting, in our opinion, of as satisfactory an explanation on the opposite supposition.

There is, we think, only one way of setting this disputed point at rest, namely, by a diligent comparison of the relative number of cases which occur among those who are in continual communication with the sick, and those who are not; other circumstances being the same, or nearly so. The observations should be made in various localities, and at different times, and the comparison should be instituted between the attendants on the sick in a cholera hospital, and the surrounding population in the *immediate* vicinity of that hospital. A very small town, or a village, is evidently the best place for such observations.

If it should be found, on comparing *a very large number* of results, that the proportion of cases among the hospital attendants is only a little greater than that among the rest of the population, such difference may fairly be attributed to the fatigue, apprehension, and confinement, which render the former more obnoxious to any prevailing disease; but, should it be found that the hospital attendants are affected in a ratio double or triple that of the other inhabitants of the place, the disease may then be set down, without hesitation, as contagious.

It is but justice to the contagionists to say, that, after the experience we have lately had of the disease in this country, their doctrine is decidedly gaining ground; but, though we ourselves incline to it, we conceive that there are hitherto no data for a positive conclusion.

There are few diseases which are, on the whole, more homogeneous in their aspect than cholera: it presents, however, occasional varieties, on which our author has some interesting remarks.

"Mr. Curtis, in his description of the disease as observed in India, mentions several varieties of it, one distinguished by the absence of vomiting and the prevalence of diarrhœa; another by the excess of vomiting and occasional absence of the dejections; and a third by very slight commotion of the system, indicated either by the evacuations or by spasm; this last, he says, he considered the least manageable of all. The form in which it most frequently presented itself to him, was that in which the alvine dejections were particularly frequent. There are, however, two other forms which this disorder has occasionally put on. One in which spasms alone have been observed, and another in which copious determinations of blood have taken place to the thorax and abdomen. Of the former of these I have met with but few examples in this country; they were, however, more frequent in India, the practitioners having repeatedly noticed this variety; and this in all probability is the convulsive disorder noticed during the prevalence of cholera, and denominated by Girdlestone and others 'idiopathic spasm.'

"But the other form, that, namely, characterized by large effusions of blood, is but rarely referred to by foreign writers, if indeed distinctly described by any, though a form which it seems to me clearly to have assumed, and is one which may perhaps throw some light on the actual nature of the complaint." (P. 17.)

Dr. Roupell alludes more fully to the last-mentioned form, when speaking of those diseases which have occasionally prevailed immediately before the invasion of epidemic cholera.

"That form of the disease which I have mentioned as indicated by discharges of blood from the mucous membrane, seemed here to be the first in advance; and if not entitled to be considered as the disease itself, might be called premonitory. Several cases, it may be recollected, excited some notice before the disease was proclaimed in London, and on the 16th of January a coroner's inquest sat on the body of a man at Shadwell, who died with urgent cramps, pain in the abdomen, and vomiting of blood. This man was a seaman, and had arrived from North Shields a week before. In this case the jury, guided by the opinion of several practitioners, some of whom had seen the cholera in India, and had been appointed to the superintendence of the metropolitan districts, came to the conclusion that the disease was not cholera. But many cases which I saw about that time and previously, occurring in persons connected with vessels trading from Sunderland, indicated a very intimate connexion with the disease in question. I may briefly mention one, which was under my own care. A man named Webster, twenty-eight years of age, sailed from Sunderland on the 20th of January, and arrived in London about the 30th. The vessel immediately obtained pratique; but a few days after, this man was seized with extreme pain in the epigastrium, the abdomen was forcibly drawn in, he had urgent vomiting, with coldness of the hands and paleness of the countenance; a warm bath being ordered and some castor oil given, the urgency of the symptoms was removed, and evacuations were produced free from any admixture of blood; the next morning an inclination to empty the bowels was felt; an enema was administered, which returned unmixed with fæcal matter; but rising to go to the chair he fainted, fell back, and died. No cause could be assigned for the attack, except some slight exposure to cold incident to a seaman's life, and which the patient in this instance seemed well able to resist, being remarkably powerful, not addicted to any excess, and living in a manner least likely to predispose to any disorder; but he mentioned that he had had a similar attack at Sunderland six weeks before. On examination after death, twenty ounces of blood, apparently venous in character, was found in the cavity of the abdomen. The peritoneum was slightly vascular. On removing the abdominal contents, and examining the intestines externally, the stomach and the small intestines to the extent of five feet were natural; when suddenly the tube was found firmly contracted, as if tied round by a thread, and from this point downwards to the ileo-colic valve the intestines were of a deep purple colour. Internally, nothing at all deviating from health was remarked in the upper part of the intestines, but below the point of stricture described the intestine was filled with dark-coloured blood, some of which was found as far as the rectum. The mucous membrane when held up to the light did not appear more vascular than usual in this part. The consistence or texture of the intestines was not altered, although they exhaled an extremely offensive odour. This case corresponded in some degree with that already alluded to at Shadwell, but differed from it in the collection of blood in the abdomen. Another example of sudden death occurred under my own observation, in the preceding December, in a man also engaged in the Sunderland trade, who was attacked with very similar symptoms, but who referred the pain more directly to the neighbourhood of the heart. By appropriate means he appeared to be relieved, yet on the morning following his admission into the hospital, on sitting up to take his breakfast, having declared he felt sufficiently well to return to his duty, he suddenly sank back, and immediately expired. The post-mortem examination shewed the small intestines and its vessels congested, some effusion of blood having taken place in the neighbourhood of the spleen, but to no considerable extent. These cases seem fairly to be attributable to the impression of the same agency which produces cholera, and in its most aggravated degree. Instances of death by hemorrhage, I know, have taken place long before the introduction of cholera maligna into our nosology; but I think the time when these happened, and the class of persons amongst whom they occurred, justify me in considering them as other than ordinary occurrences." (P. 32.)

Dr. Roupell considers the treatment of cholera under the heads of Bleeding, Emetics, Salines, Stimulants, Purgatives, Sedatives.

*Bleeding*. Our author has found no advantage from this remedy in the advanced stage of cholera. He combats the idea that bleeding can act, as supposed by many, simply by disencumbering the circulation. An instance of its failure, when employed with this view, is given in the following case.

"Edward Hide, of temperate habits, and having previously enjoyed good health, was admitted into St. Bartholomew's cholera hospital, on the 10th of September. At that time his countenance was anxious, his skin, tongue, and breath, were cold; his hands felt clammy, his fingers were corrugated; he was tormented by intense thirst, urgent vomiting and severe cramps, and his stools were frequent, copious, and like rice-water. Warmth and stimuli failed to rouse him. On this a vein was opened. The blood flowed slowly, was thick, and dark-coloured. No strength was acquired while the blood flowed, no rise in the pulse took place, indeed, he seemed to be sinking so fast under the remedy, that I deemed it prudent to stop when but a small quantity had flowed. His symptoms increased, and he died thirty-six hours after admission. This case I cite as one of many in which a similar result has followed." (P. 42.)

Our own experience coincides with this. We have never seen bleeding practised but in the stage of collapse, in which it had either no effect at all, or accelerated the death of the patient. It is also to be remembered, that bleeding in the advanced period of cholera is not always a remedy of possible application; for those who have tried the experiment know well that, in a considerable proportion of cases, little or no blood can be obtained. On opening a vein, nothing issues but a tablespoonful or two of a black, cold, semigelatinous fluid.

"But venesection," observes our author, "nevertheless, has done good in cholera. It must then operate beneficially in some way, though certainly not by relieving the circulation mechanically. Can it be by arresting the diarrhœa? An answer to this question may perhaps be furnished by the following narration.

"James Ward, æt. fifty-six, had been for some months a patient under my care, for a cerebral affection, on board the Dreadnought, the Seaman's Hospital. On the 21st of September he was attacked with active purging. An emetic was administered, and calomel and opium given every four hours. Neither the one nor the other checked the diarrhœa, which continued for three days, when I ordered him to lose sixteen ounces of blood. The diarrhœa stopped immediately, and without further treatment the patient recovered from this attack in the bowels. It appears then that venesection in this case arrested the diarrhœa; and it is probable that this diarrhœa was premonitory of cholera, as that disease was then prevailing on board the hospital ship, and a man had died of it in the adjoining bed.

"Robert Blair, æt. thirty-two, also a patient on board the Dreadnought, had been admitted for deafness, and was attacked with violent purging on the night of the 12th of September. He applied for relief on the 13th, and then stated, that although violently seized the night before, yet that for two days previously his bowels had been relaxed. His tongue at the time of his application was pale and cold, his eye looked dark and sunken, he complained of a sense of constriction in his chest, his pulse was labouring and irregular, he was constantly purged, and his stools were watery and copious. Sixteen ounces of blood were taken from his arm, an emetic was afterwards given; the purging which had previously occurred every half hour then ceased; no other remedy was employed, and he was discharged well of his recent attack. In this case also it appeared that venesection arrested the diarrhœa, and that when the tongue was pale and cold, the eye dark and sunken, and the pulse feeble and irregular; here again there was great suspicion that this diarrhœa was the diarrhœa of cholera. Strong as the belief might be that the cases which I have cited were referable to cholera, yet in them the characteristic discharges were absent. But I will briefly mention one of a person of the name of Powell, thirty years of age, who consulted me on the 29th of August, about the middle of the day: he had been much exposed to the contagion, as he was assistant to the undertakers of St. Bartholomew's Hospital. He complained of vomiting, purging, and cramps. For three previous days he had been troubled with diarrhœa. Five grains of calomel with a grain of opium were given immediately, and repeated in four hours. When visited in the evening, the purging was incessant, the evacuations gruelly with small white flakes, but his skin was still warm, and his pulse had not yet sunk. Twenty ounces of blood were drawn from his arm, and a scruple of Hyd. c. cret. were given; the diarrhœa at once ceased, and he passed a good night. Purgative medicines were afterwards required: the case terminated favourably, and the man was discharged well on the fifth day. This case was one undoubtedly of cholera. A large venesection was practised, and the immediate and unequivocal effect was to arrest the diarrhœa. These cases are far from solitary, but were selected merely for the sake of illustration. The inferences which I draw from them are:

"1. That venesection will stop a diarrhœa, when calomel and opium and an emetic have failed.

"2. That venesection will stop the purging in cholera before collapse has taken place.

"3. That in collapse, bleeding is a remedy of doubtful benefit, if not positively hurtful." (P. 43.)

What power bleeding may have over the diarrhœa of cholera, we can estimate only from the reports of others; but that it will often stop a common diarrhœa, we know from repeated experience. Indeed, where the diarrhœa is obstinate, and the patient moderately robust, we have sometimes found venesection the most efficacious of all remedies.

Dr. Roupell gives a case illustrative of the power of bleeding over the spasmodic symptoms of the disease:

"Before I quit the subject of the benefit to be derived from bleeding, the chief of which I have endeavoured to shew to be in checking the diarrhœa, I will mention a case which was admitted into St. Bartholomew's cholera hospital, in which spasms alone were the symptom of the disease.

"Emma Lloyd, æt. thirty, applied for relief at ten o'clock in the morning of the 1st of September, labouring under frightful spasms, which came on in paroxysms about every five minutes: they commenced in the abdomen, drawing the muscles into hard masses; the muscles of the chest then suffered, and the fit terminated by a forcible retraction of the head, producing sometimes complete opisthotonos. The pain occasioned was extreme. The pulse was natural; neither vomiting nor purging was present. No obvious cause could be assigned for the attack, which began on the same morning. She was not hysterical, and had never had fits of any sort. She was bled to ten ounces, when the spasms immediately ceased, and did not again recur. The next object was to open the bowels, for which purpose appropriate medicines were directed. The symptoms which then presented themselves were vomiting and the most intense thirst: these were, however, relieved by the operation of the remedies already mentioned, but continued slightly for two days, when they left her entirely.

"This I consider to be one of those mentioned by Indian practitioners as 'spasm,' and of which I met with several in a milder form during the summer of 1832. My opinion was undecided when first I saw this case, but the course which it afterwards assumed, the vomiting, and thirst, seemed to connect it intimately with the disorder prevailing at that time. My belief then is, that bleeding is an important remedy in the more severe forms of the disease, either in the stage of premonitory diarrhœa, or even when it has more clearly developed itself, if resorted to before the system is exhausted by copious discharges." (P. 49.)

*Emetics.* After venesection Dr. Roupell ranks emetics, observing, however, with truth, that "there are few medical men, who have watched the cholera even in its later periods, who have not been deluded into false hopes from the obvious improvement occasioned by the full effect of vomiting. In fact, the pulse will rise, the lividity of the skin will for a while disappear, but, with other unfavourable signs, in a short time recur." (P. 50.) He considers emetics as a useful means of rousing the powers of the system after the diarrhœa has been checked by bloodletting; he adds, that "those cases in which vomiting is the chief symptom, and is very urgent, may be brought successfully to a close by keeping up the action of the stomach with copious draughts of cold water, to which class of cases that simple mode of treatment seems the most applicable. I had one case under my care which terminated favourably, in which nothing else was taken in the earlier period of the disease." (P. 53.)

*Salines.* On Dr. Stevens's saline treatment our author remarks:

"I felt desirous of trying the method which he recommended, but I could not bring my mind to a conclusion as favourable as Dr. Stevens has done. I either found no benefit from it, or could not persist in its employment; the patients growing worse, or positively refusing to swallow the powders, from their causing immediate and almost incessant vomiting." (P. 55.)

Dr. Stevens's *cases* we do not consider worthy of the smallest attention, as it is pretty clear that this gentleman either grossly deceived himself, or wilfully and knowingly — &c. We are not fond of uncharitable conclusions. To the theory promulgated by Dr. Stevens we shall presently have occasion to recur.

In speaking of the saline *injection*, Dr. Roupell observes, that he has seen it "accelerate the fatal termination of some cases, and prove the cause of death in others, by overloading the brain, and occasioning effusion into the ventricles. Still its good effects are sometimes magical, and shew that many of the symptoms in cholera arise from causes that can be easily remedied; but its frequent failure proves that more than the mere supply of salts is requisite to remove the disease." (P. 57.)

It appears to us that the benefit, either temporary or permanent, which has unquestionably followed the use of venous injection in some cases, has been too hastily ascribed to the chemical properties of the injected fluid: it is, we think, more probable that this remedy acts simply as a stimulus applied to the internal membrane of the heart and blood-vessels; it may also act beneficially by diluting their inspissated contents, at the same time that its stimulating properties, and the distension which it occasions, rouse the vessels to their wonted action. All this, however, is mere hypothesis.

*Stimulants.* On this head our author's observations are very general. With respect to brandy, æther, and the essential oils, he thinks that they "can only with safety be resorted to at the commencement of the disease, and then undoubtedly they have, in some instances, completely put a stop to all those indications which are premonitory of cholera." (P. 60.)

Of the power of the cold affusion in rousing the powers of the system, Dr. Roupell states that he has had repeated experience; and from no remedy has he seen such decided amendment in the stage of collapse, except the full effect of emetics.

*Purgatives.* These he believes to be among the most important remedies in cholera.

"But the term 'purgative' must be limited in its meaning, and all cathartics are by no means to be recommended. I have seen a colocynth pill, taken to remedy an obstruction in the bowels during the prevalence of cholera, excite a diarrhœa which never ceased until life itself was exhausted. I have seen active purges themselves produce the disorder; and those which occasion copious serous evacuations are under no circumstances to be administered. Such as act simply by removing the irritating contents of the intestinal canal may be directed, and such as combine with purgative effects an astringent character I have generally ordered: rhubarb, for instance, either combined with, or given shortly after, a large dose of calomel. But purgative medicines, I should say, are chiefly to be resorted to early in the disease." (P. 61.)

*Narcotics.* Of these our author only alludes to opium, which he considers as useful only at the commencement of the disease.

"If opium can (as has been asserted) in all cases restrain the discharges, I am persuaded it will often fail to do so, unless given in doses so large as to hazard the safety and life of the patient; at the same time that, if properly administered at the commencement, it is of more value than any other remedy; but the point of time at which alone it can be properly employed must be before those actions are established which directly tend to depress the system. It should be given in full doses, in order that the effect may be speedily produced; and, if not at once beneficial, bleeding and other means should be resorted to without delay." (P. 62.)

In his concluding lecture Dr. Roupell takes a judicious survey of the pathology of cholera. To the theory which attributes the disease to a chemical change in the circulating fluids, he properly opposes the suddenness with which a patient is sometimes attacked, and the rapidity with which he recovers; and the experiments of Dr. Christie, which go to prove that, at the commencement of cholera, the blood has not undergone any alteration. The former objection is, we think, quite conclusive against Dr. Stevens's theory. When, as often happens in India, and sometimes elsewhere, a man previously in perfect health is struck down by cholera in an instant, cold and pulseless, where are his salts gone to?—have they flown into the air? And, when a man so affected gets well almost as fast as he got ill, where do the salts come from? Do they arrive on the wings of the wind, and rush into his veins all of a sudden?

That rapid chemical changes may take place in the constitution of the blood, we see no reason to doubt; but that all its saline ingredients should be abolished in half a minute, and regenerated in half an hour, passes all understanding. It may be said that we are supposing an extreme case; true: but what sort of pathological theory is that which will not apply to the most strongly marked and concentrated case of the disease it is intended to explain? It is the very case to which it ought to apply most emphatically.

To the views of those who consider inflammation or congestion in the mucous membranes as the proximate cause of cholera, Dr. Roupell opposes the results of his dissections:

"In general, I may say, in the most rapid cases which fell under my own observation, that the mucous membranes were pulpy, white, not loaded with blood, nor were the neighbouring vessels unusually distended; the venous and arterial branches of the mesentery, on the contrary, were not unfrequently empty, or nearly so." (P. 70.)

The following is a general summary of his post-mortem investigations:

"The appearances discovered after death in the bodies of those who have died of cholera, are dryness of all the serous membranes, from the want of their usual lubricating secretions; absorption rapidly going on, so that the features are so changed in a few hours that persons have been unable to recognise their own relations. The secretions appear to be all suspended. These consequences cannot be maintained to be the ordinary result either of hemorrhage, inflammation, or congestion. I have examined with much attention the inner linings of the arteries, and of the veins of the abdomen and have traced their ramifications as far as instruments would serve me, and the eye could follow them, but without being able to discern any morbid change. The thoracic duct when I have examined it has been empty. The most constant appearance which I have noticed, has been a peculiar redness of the external coat of the intestines, a redness of a rosy hue, peculiar in its tint, and obviously owing to some change in the capillary arteries, differing entirely from the colour of the blood in the veins, and I have also noticed the same aspect in the transparent vessels of the peritoneum." (P. 73.)

Dr. Roupell gives an interesting analysis of the blood, bile, and urine, in a case which terminated fatally in the stage of consecutive fever. For this analysis he expresses himself obliged to Dr. O'Shaughnessy: it presents the novel feature of the existence of urea in the bile.

The blood contained in the 1000—

| | | |
|---|---|---|
| Water | . . . | 702.00 |
| Urea | . . . | 3.66 |
| Salts | . . . | 6.00 |
| Albumen<br>Fibrine<br>Oily matter<br>Hæmatosine | . . } . | 288.34 |

The urine contained—

| | | |
|---|---|---|
| Water | . . . | 973.75 |
| Urea | . . . | 4.40 |
| Albumen | . . . | 19.35 |
| Salts | . . . | 2.50 |

And a trace of uric acid.

The bile, which had been evacuated in great quantity during life, both by vomiting and stool, and which presented nothing peculiar in its appearance, contained six parts in the thousand of salts, and three parts of *urea*. (P. 84.)

After demolishing other people's theories of cholera, Dr. Roupell introduces one of his own, in which he maintains "that the primary cause of the effects witnessed in that disease arises from an affection of the nervous system; that the immediate result of this is an irritability of the lymphatic portion of the vascular system, occasioning increased fluid discharges, which exhaust the system, and prove eventually fatal." (P. 74.)

With respect to the primary affection of the nervous system, we are inclined to agree with our author: of his theory in general, we have only to say, that it appears to us to be as good, and no better, than some which have preceded it; but for its full exposition we must refer to Dr. Roupell's book. The fact is, we are heartily tired of theories of cholera, and have no doubt that the greater part of our readers participate in our weariness. It seems to us that, in the investigation of obscure diseases, to whose pathology we have no rational clue, we are less profitably occupied in forming conjectures on their proximate cause, than in comparing them with those maladies to which they seem, from their symptoms, to have a natural affinity, in the hope that they may eventually throw some light on each other.

There is, we conceive, only one diseased state to which malignant cholera, generally speaking, bears the smallest resemblance, namely, asthenic fever; and, if we contrast cholera with fever, each in their most intense form, we shall observe several very striking points of similarity. We quote the following description of fever, when fatal at its first onset, from Fordyce, whose work, though not recent, is, in our opinion, the best and most philosophical that has yet appeared on the subject of fever.

"When the attack is fatal it sometimes kills in five minutes, sometimes it requires half an hour, seldom longer than that time. While the patient is yet sensible, violent headach with great sense of chilliness take place, the extremities become very cold and perfectly insensible; there is great prostration of strength, so that the patient is incapable of supporting himself in an erect posture; he becomes pale, his skin of a dirty brown, and he is soon insensible to external objects; the eyes are half open, the cornea somewhat contracted. If the patient goes off very soon, the pulse is diminished, and at last lost, without frequency taking place, but if it be long before he dies, the pulse becomes excessively small and frequent, all the appearances of life gradually subside, and the patient is carried of." (*Dissertations on Fever*, vol. i. p. 181.)

Now, if such a case be compared with cholera in its most concentrated form, that in which there is neither vomiting, purging, nor spasms, but sudden arrest of the circulation, and extinction of the powers of life, these two forms of disease, though certainly distinct, and in some respects widely different, still present a generic similarity which might entitle them to be considered as cognate; and a knowledge of the real cause of either of them would probably enable us to reason more satisfactorily on that of the other.

Dr. Armstrong considers cholera as a form of what he calls *congestive fever;* and Mr. Searle is of the same opinion. Without precisely adopting this view of the subject, we think the analogy is so strong that it ought by no means to be lost sight of. The relationship of cholera to fever is also countenanced by the very frequent instances in which the former, having passed through its cold stage, is actually developed into a fever of the typhoid type; though it is admitted that this fever appears to be one *sui generis.*

Of one thing we are certain, that we have seen instances of malignant cholera, both in the cold stage and that of reaction, which (putting aside the history of the case, and judging merely from the symptoms as they presented themselves,) would at once have been referred to the corresponding stages of typhus.

Dr. Roupell, in his concluding remarks, thus adapts his treatment to the views which he entertains of the nature of the disease:

"In the premonitory stage, calomel and opium, brandy and other stimulants, may be exhibited, when only the nervous extremities are affected, or determination be only slightly occasioned; this may blunt the impressions, and thus render the nerves insensible to the cholera stimulus, or, by general excitement to the sanguiferous system, the disease may be arrested. Should, however, this determination continue, bleeding must be had recourse to, and this I conceive to act by emptying the vessels and allowing the increased discharge from the lymphatics to circulate through the veins, instead of being poured out through the bowels; but bleeding will by no means succeed if late resorted to, when the blood is thickened, and has lost ingredients which are essential to its circulation and to the vital processes; at that time any diminution of its quantity gives no relief, indeed seems to hasten the fatal result. In complete collapse, emetics and the cold affusion are infinitely the most powerful stimuli, and when the disease is once arrested, salines, I conceive, may be beneficial; opium in very small doses may also be of use to check the irritability of the stomach; but in the more advanced stages, when the head is bewildered, and when there is a disposition to coma, it is under no circumstances to be employed. I imagine that the saline injection does good, if the lost constituents of the blood be supplied in those cases in which exhaustion has been rapid, and the disease is disposed to cease; but it is a remedy far from innocent in its nature, and it is to be borne in mind, that it can do harm as well as good; one objection to its use is, that it terminates often in phlebitis, but this may be obviated by gentleness in performing the operation, and by using a small flexible elastic tube." (P. 88.)

Our author has great confidence in the resources of art, when employed in the commencement of cholera; "for," says he, "I have rarely found any case, in which the disease was presented to me in its early stage, which did not yield successfully to medical treatment."

We are happy that Dr. Roupell's experience has been so encouraging: we cannot say that our own has been equally so, even when the disease came early under treatment. Still there is no doubt that active and judicious practice will accomplish much at this period, and that a great many cases, perhaps the larger proportion, will be saved. If indeed by the early stage of cholera the precursory diarrhœa only be meant, we agree with Dr. Roupell, that it is very much under the control of medicine; but we must not absolutely call checking a watery diarrhœa curing cholera, because we cannot be certain, in any given case, that such diarrhœa was really the forerunner of that malady.

With regard to the accounts published by various individuals of the wonderful success of treatment in the worst and pulseless stage of cholera, we say, without the smallest ceremony, that we do not believe them. We have seen many such cases treated, and we have treated some ourselves; the treatment was various, but the result uniform: we never saw a single pulseless patient recover under any treatment whatsoever. That such patients do sometimes recover, we have not the least doubt; but, as our observations were not confined to one locality, or one mode of treatment, but extended to several districts, and great variety of treatment, we must be excused if we are more prone to believe our eyes than our ears; and if, in opposition to "undoubted testimony," we regard recovery from the worst stage of cholera as a rare occurrence, and the powers of medicine in this case as nearly nugatory.

In his enumeration of remedies, Dr. Roupell only slightly alludes to *mercury*, under the head of stimulants; he afterwards, however, expresses his belief of the inefficacy of that remedy, mentioning one case in which collapse actually supervened while the mouth was affected. Our own observation agrees with his: we have seen calomel given in small and in large doses, but never with any apparent benefit. In the very last case that came under our own treatment, we gave it in doses of a drachm every hour, from which administration we could not perceive the smallest effect of one kind or another: the patient died.

If we have insisted somewhat on so disheartening a subject as the inefficacy of medicine in the treatment of cholera, it has only been with the wish of counteracting, as much as in us lies, that abject spirit of credulity which is the bane of our art, and which so often exposes its professors to ridicule. If a man has a tale to tell that would be laughed at in a nursery, he has only to promulgate it in a medical society, to obtain credence from somebody.

In taking leave of Dr. Roupell, we may just be permitted to suggest that, in treating of medical subjects, it is by no means essential, though extremely fashionable, to abjure the use of the English language. These lectures abound with such sentences as the following:

"Not less various were the remedies, or more contrary in their tendency the plans of treatment recommended for internal administration." (P. 40.)

"This person's mode of life, and her condition preceding the attack induced a decidedly unfavorable prognosis, which the character of symptoms shewed to be of the most malignant type." (P. 53.)

Those who are accustomed to see patients swallow *plans* of treatment, or to determine the *type* of a *prognosis*, may make English of these sentences; but we cannot.

On the whole, Dr. Roupell's is one of the best books that has been published on cholera. It is written with great candour and good sense, and contains more original matter than might have been expected in treating of a subject on which Medicine has expended all her resources, and, we may add, Fiction not a few of her embellishments.

